# Clinical and Echocardiographic Factors Associated With Left Ventricular Thrombus Recurrence

**DOI:** 10.1016/j.jacadv.2026.102873

**Published:** 2026-06-17

**Authors:** Kirsten M. Lipps, Hossam Elbenawi, Samuel Heller, Robert D. McBane, Waldemar E. Wysokinski, Ana I. Casanegra, Stanislav Henkin, Thom W. Rooke, Paul W. Wennberg, David A. Liedl, Raymond C. Shields, Damon E. Houghton

**Affiliations:** a Division of Vascular Medicine, Department of Cardiovascular Medicine, Mayo Clinic, Rochester, Minnesota, USA; b Second Faculty of Medicine, Charles University, Prague, Czech Republic; c Division of Hematology, Department of Internal Medicine, Mayo Clinic, Rochester, Minnesota, USA

**Keywords:** echocardiography, heart failure, ischemic cardiomyopathy, left ventricular aneurysm, myocardial infarction

## Abstract

**Background:**

Left ventricular (LV) thrombus (LVT) recurrence risk influences decision-making regarding oral anticoagulation (OAC) duration.

**Objectives:**

The aim of the study was to evaluate risk of LVT recurrence and factors associated with recurrence.

**Methods:**

This was a multicenter, retrospective cohort of patients with resolved LVT, diagnosed by echocardiography. Clinical and echocardiographic characteristics at LVT diagnosis and resolution were evaluated for association with recurrence. Multivariable Cox proportional hazards regression was used to identify determinants of LVT recurrence.

**Results:**

Among 252 patients (mean age 64.5 years, 78.6% male), LVT recurred in 36 (14.3%). Although LV systolic function at the time of presentation was similar between those with and without recurrence, patients who developed recurrent LVT had lower LV ejection fraction (LVEF) (34.0% vs 40.0%; *P* = 0.04) and more frequently had LV apical aneurysm (25.0% vs 6.5%; *P* = 0.001) at LVT resolution. Continuing OAC after LVT resolution was associated with less recurrence (54.7% vs 25.0%; *P* = 0.005). In multivariable analysis, LV apical aneurysm (HR: 3.12; 95% CI: 1.43 to 6.84) and previous ischemic stroke (HR: 2.75; 95% CI: 1.22-6.17) were associated with higher rates of recurrence, whereas higher LVEF (HR: 0.96 per 1% increase; 95% CI: 0.95-0.99) and OAC continuation (HR: 0.27; 95% CI: 0.13-0.58) were associated with decreased recurrence.

**Conclusions:**

In this population, LVT recurred in 14% of patients and was associated with LV apical aneurysm and prior stroke, whereas lower rates of recurrence were associated with higher LVEF and continuation of OAC. Echocardiographic characteristics at the time of LVT resolution were associated with LVT recurrence.

Left ventricular (LV) thrombus (LVT) is a serious complication, which can occur following acute myocardial infarction^1^ and in patients with non–ischemic cardiomyopathy.^2^ With contemporary oral anticoagulation (OAC) therapy, LVT can resolve in up to 90% of patients, with higher rates observed in those treated longer.^3^, ^4^, ^5^ Multiple guidelines recommend anticoagulation therapy for patients with LVT, with experts suggesting a minimum of 3 to 6 months of treatment when LVT occurs following acute myocardial infarction.^6^, ^7^, ^8^ Less data exist to guide treatment of LVT associated with other etiologies of LV systolic dysfunction.^6^^,^^9^, ^10^, ^11^

LVT recurrence has been observed in up to 25% of patients in small studies, leading to concern about whether anticoagulation therapy can be safely discontinued after LVT resolution and emphasizing a need to better understand parameters affecting this risk.^12^, ^13^, ^14^, ^15^, ^16^, ^17^, ^18^, ^19^ For those with LV apical aneurysm or hypercoagulability, the rate of LVT recurrence is even higher.^12^, ^13^, ^14^, ^15^, ^16^, ^17^, ^18^, ^19^ The purpose of this study was to evaluate the incidence of LVT recurrence among a large cohort of patients with LVT resolution and identify clinical and echocardiographic factors associated with recurrence. These data will help inform decision-making regarding the duration of OAC following LVT resolution.

## Methods

### Patient population

This retrospective cohort study was approved by the Mayo Clinic Institutional Review Board as minimal risk for patients who had previously consented to have their medical records included in observational research, and it was conducted in accordance with the Declaration of Helsinki. We included all adults (≥18 years), regardless of underlying cardiovascular history, who had evidence of LVT as diagnosed by echocardiogram performed at any of the three Mayo Clinic academic campuses or associated satellite community-based locations between January 2018 and July 2023. We did not use other imaging modalities, such as cardiac magnetic resonance imaging (CMR) or cardiac computed tomography, to identify potential patients. A natural language processing (NLP) algorithm was developed to screen echocardiogram reports for the presence of LVT.^20^ An initial derivation cohort of 100 echocardiogram reports was used to design the algorithm; this was then evaluated in a validation cohort of 300 separate echocardiogram reports that had previously been classified by manual review. Within the validation groups, the NLP algorithm had 85% sensitivity and 100% specificity for identifying LVT within the echocardiogram reports.

The NLP algorithm was then used to identify echocardiogram reports of patients who potentially had LVT, and these reports were then manually reviewed to ensure the presence of LVT. We then independently reviewed echocardiographic images from a subset of the final cohort (>50%) to confirm the findings of LVT, and the specificity on this review was 100% for the final cohort identified by the NLP. We did not review any echocardiographic images of patients not identified by the NLP. We included only patients who were initiated on a vitamin K antagonist or direct oral anticoagulant medication within 30 days of LVT diagnosis and who had at least one follow-up echocardiogram that documented LVT resolution at least 30 days after LVT diagnosis (Supplemental Figure 1). We included only patients with resolved LVT, excluding 19.2% of patients with persistent LVT. We also excluded patients with chronic LVT (identified by echocardiogram performed before January 2018), as these thrombi were considered less likely to resolve with anticoagulation. Finally, we excluded patients treated with anticoagulation in the 7 days preceding LVT diagnosis, as these cases were considered to represent anticoagulation failure.

### Data collection

Demographic and clinical data at the time of LVT diagnosis were manually abstracted from the electronic medical record for a baseline comparison between patients with LVT recurrence and those without. Clinical data included cardiovascular comorbid conditions, renal function, hematological parameters, and prothrombotic risk factors.

Echocardiographic reports at LVT diagnosis and resolution were manually reviewed to abstract data regarding LV systolic function, including LV ejection fraction (LVEF) and segmental wall motion (ie, normal, global hypokinesis, or regional wall motion abnormalities). For patients with LV apical wall motion abnormalities, we further specified whether segments were classified by the interpreting echocardiographer as hypokinetic, akinetic, dyskinetic, or aneurysmal. The use of ultrasound enhancing agents during echocardiography was not universal. An LV apical aneurysm was defined as a thin, scarred region of myocardium, which was dyskinetic throughout the cardiac cycle.

Data regarding the use of OAC and antiplatelet therapies, beginning at the time of LVT resolution, were manually abstracted from the electronic medical record. Anticoagulation therapy was categorized as “Uninterrupted OAC” or “Interrupted/Discontinued OAC.” The “Uninterrupted OAC” group was defined by near continuous OAC therapy from the time of LVT resolution until death or the end of the follow-up period, aside from brief interruptions in therapy of < 14 consecutive days. In contrast, the “Interrupted/Discontinued OAC” group included patients who had prolonged interruption in therapy of at least 14 consecutive days in duration or who discontinued therapy altogether at some point after LVT resolution.

### Outcomes

The primary outcome of interest was the incidence of LVT recurrence. This was defined by the presence of at least one new LVT documented within an echocardiogram report after echocardiographic evidence of initial LVT resolution. To identify LVT recurrence, all available echocardiogram reports in the electronic medical record following initial LVT resolution were manually reviewed. Secondary outcomes included composite and individual incidences of stroke and systemic embolism; all-cause mortality; and major bleeding, from the time of LVT resolution until the end of the follow-up period. Major bleeding was defined according to the International Society on Thrombosis and Haemostasias guidelines.^21^ Secondary outcomes were confirmed through manual review of electronic medical record documentation. Patients were followed from the time of echocardiographic resolution of the initial LVT until death or September 2024, whichever occurred first.

### Statistical analysis

Continuous variables are described as mean (standard deviation [SD]) or median (25th percentile-75th percentile [Q1-Q3]), whereas categorical variables are reported as counts and percentages. Time-to-event outcomes were analyzed using the Kaplan-Meier method (death) or Aalen-Johansen estimator (death is a competing risk) and comparisons made using log-rank or Gray test, respectively. Cumulative incidence estimates are provided with 95% confidence intervals (CI). A multivariable cause-specific Cox proportional hazards regression analysis was conducted to identify independent predictors of LVT recurrence with results presented as hazard ratios (HR) with 95% CI. Variables included in the models were selected based on clinical relevance and statistical significance in univariable analysis. To reduce overfitting in the setting of a small number of LVT recurrence events during the follow-up period, two Cox models were developed: Model 1 (“Uninterrupted OAC”, LVEF, ischemic cardiomyopathy [ICM], and LV apical aneurysm) and Model 2 (“Uninterrupted OAC”, prior ischemic stroke, and hereditary thrombophilia). To address concerns about competing risks, we performed multivariable regression using the Fine and Gray subdistribution hazards model to evaluate the association between covariates and the cumulative incidence of recurrent LVT, while accounting for death as a competing event. Subdistribution HRs with 95% CI were estimated. Statistical analyses were performed using JMP software (version 17.2; JMP Statistical Discovery LLC, 2022) or BlueSky Statistics (version 10.3.4; BlueSky Statistics, 2024).

## Results

### Patient population

Between January 2018 and July 2023, 312 patients had LVT identified by echocardiography, and 60 (19.2%) were excluded due to lack of resolution. The remaining 252 had echocardiographic evidence of LVT resolution and were included in the analysis (Supplemental Figure 1). Among included patients, the mean (SD) age at LVT diagnosis was 64.5 (15.0) years, 198 (78.6%) were males, and 230 (91.3%) were White. The median (Q1-Q3) time to echocardiographic evidence of initial LVT resolution was similar between patients who had LVT recurrence and those who did not during the follow-up period (103 [72-322] vs 103 [70-193] days; *P* = 0.66).

### Demographic and clinical characteristics

Demographic and clinical characteristics at LVT diagnosis were compared between patients with and without LVT recurrence (Table 1). Previous ischemic stroke showed a trend toward higher prevalence among patients with LVT recurrence compared to those without recurrence (22.2% vs 9.7%; *P* = 0.08) whereas most other comorbidities were similar between the groups. Overall, 37 of 252 (14.7%) patients had history of arterial thromboembolism, and 24 (9.5%) had prior venous thromboembolism; the prevalence of these comorbidities were not significantly different between the groups.

**Table 1:** Demographic and Clinical Characteristics at LVT Diagnosis, Stratified by LVT Recurrence

	Total (N = 252)	No LVT Recurrence (n = 216)	LVT Recurrence (n = 36)	HR (95% CI)	*P* Value
Age, mean (SD), years	64.5 (15.0)	64.0 (15.0)	67.0 (14.0)	1.01 (0.99-1.04)	0.21
Male, n (%)	198 (78.6)	167 (77.3)	31 (86.1)	1.82 (0.71-4.69)	0.21
White race, n (%)	230 (91.3)	199 (92.1)	31 (86.1)	0.55 (0.21-1.41)	0.21
Acute coronary syndrome, n (%)	157 (62.3)	130 (60.2)	27 (75.0)	1.85 (0.87-3.95)	0.11
STEMI, n (%)	79 (31.3)	67 (31.0)	12 (33.3)	0.96 (0.48-1.91)	0.90
NSTEMI, n (%)	49 (19.4)	41 (19.0)	8 (22.2)	1.38 (0.63-3.02)	0.43
Unstable angina, n (%)	3 (1.2)	3 (1.4)	0 (0)	-	-
Ischemic cardiomyopathy, n (%)	162 (64.3)	132 (61.1)	30 (83.3)	3.10 (1.29-7.44)	0.01
Ischemic stroke, n (%)	29 (11.5)	21 (9.7)	8 (22.2)	2.03 (0.93-4.47)	0.08
Carotid artery stenosis, n (%)	7 (2.8)	6 (2.8)	1 (2.8)	0.83 (0.11-6.10)	0.86
Peripheral arterial disease, n (%)	19 (7.5)	16 (7.4)	3 (8.3)	1.30 (0.40-4.25)	0.66
Atrial fibrillation/flutter, n (%)	53 (21.0)	44 (20.4)	9 (25.0)	1.47 (0.69-3.12)	0.32
Ventricular tachycardia, n (%)	29 (11.5)	25 (11.6)	4 (11.1)	1.05 (0.37-2.96)	0.93
Myocarditis, n (%)	20 (7.9)	19 (8.8)	1 (2.8)	0.35 (0.05-2.52)	0.30
Hypertension, n (%)	184 (73.0)	155 (71.8)	29 (80.6)	1.64 (0.72-3.74)	0.24
Dyslipidemia, n (%)	184 (73.0)	155 (71.8)	29 (80.6)	1.53 (0.67-3.50)	0.31
Diabetes mellitus, n (%)	85 (33.7)	76 (35.2)	9 (25.0)	0.70 (0.33-1.50)	0.36
Current tobacco use, n (%)	60 (23.8)	50 (23.1)	10 (27.8)	1.15 (0.56-2.39)	0.70
Previous tobacco use, n (%)	82 (32.5)	70 (32.4)	12 (33.3)	1.04 (0.52-2.07)	0.92
Chronic kidney disease, n (%)	84 (33.2)	74 (34.3)	10 (27.8)	0.65 (0.16-2.71)	0.55
Stage 3 (eGFR 30-59 mL/min/1.73 m^2^), n (%)	78 (30.8)	69 (31.9)	9 (25)	0.77 (0.36-1.65)	0.51
Stage 4 (eGFR 15-29 mL/min/1.73 m^2^), n (%)	2 (0.8)	2 (0.9)	0 (0)	-	-
Stage 5 (eGFR <15 mL/min/1.73 m^2^), n (%)	4 (1.6)	3 (1.4)	1 (2.8)	1.50 (0.20-10.93)	0.69
Previous arterial thromboembolism, n (%)	37 (14.6)	28 (13.2)	9 (25)	1.82 (0.86-3.88)	0.12
Previous venous thromboembolism, n (%)	24 (9.5)	21 (9.7)	3 (8.3)	0.91 (0.28-2.96)	0.87
Antiphospholipid syndrome, n (%)	2 (0.8)	2 (0.9)	0 (0)	-	-
Hereditary thrombophilia^a^, n (%)	5 (2.0)	3 (1.4)	2 (5.6)	3.01 (0.72-12.58)	0.13
Erythrocytosis/polycythemia, n (%)	17 (6.7)	13 (6.0)	4 (11.1)	1.62 (0.57-4.58)	0.36
Arterial embolism at LVT diagnosis, n (%)	31 (12.3)	28 (13.0)	3 (8.3)	0.62 (0.19-2.03)	0.43

eGFR = estimated glomerular filtration rate; LVT = left ventricular thrombus; NSTEMI = non–ST-segment elevation myocardial infarction; STEMI = ST-segment elevation myocardial infarction.

a Includes Factor V Leiden, Prothrombin gene mutation, Protein S deficiency, Protein C deficiency, and antithrombin deficiency.

ICM was more prevalent among patients with LVT recurrence compared to those without (83.3% vs 61.1%; *P* = 0.01) (Table 1). The frequency of dilated cardiomyopathy was not significantly different between the groups (16.7% LVT recurrence vs 22.2% no recurrence; *P* = 0.30) (Supplemental Table 1). The remainder of heart failure etiologies was uncommon and not significantly different between the groups (Supplemental Table 1).

### Echocardiographic characteristics

**Figure 1:**
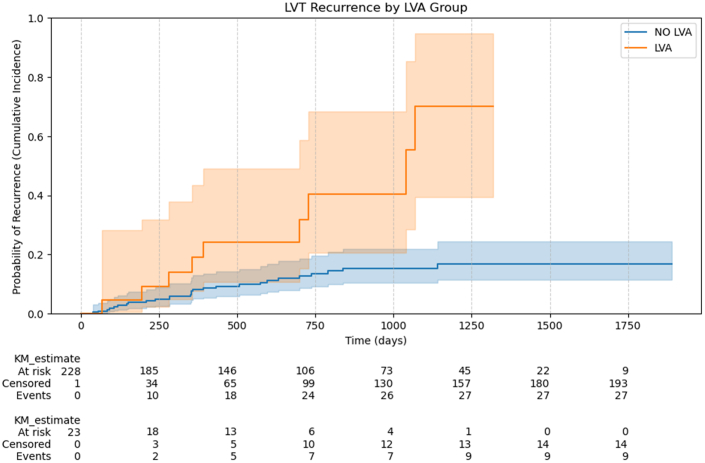
**Cumulative Incidence of LVT Recurrence, Stratified by the Presence of Left Ventricular Apical Aneurysm** Left ventricular apical aneurysm after LVT resolution was associated with a significantly increased risk of LVT recurrence during the follow-up period. KM = Kaplan Meier; LVA = left ventricular aneurysm; LVT = left ventricular thrombus.

The mean (SD) LVEF at LVT diagnosis was 30.5% (13.4%), and this was not significantly different between patients with LVT recurrence and those without (*P* = 0.87) (Table 2). However, at LVT resolution, the mean (SD) LVEF was lower in patients with LVT recurrence (34.0% [12.4%] vs 40.0% [15.3%]; *P* = 0.04) (Table 3). At LVT diagnosis, only 2 patients (0.8%) had LV wall motion abnormalities in segments other than the apex (ie, normal LV apical function); these patients did not experience LVT recurrence. In contrast, at LVT resolution, 33 patients (13.0%) had normal LV apical systolic function with wall motion abnormalities in other LV segments; this was not significantly different between groups (8.3% LVT recurrence vs 13.9% no recurrence; *P* = 0.33). The LV apical systolic function was not significantly different between groups at LVT diagnosis; 12 patients (4.8%) had a LV apical aneurysm at LVT diagnosis, and the prevalence was not significantly different between groups. The number of segments involved in the LV apical aneurysm was also not different between groups at LVT diagnosis. However, at resolution, patients with LVT recurrence had a significantly higher prevalence of LV apical aneurysm (25.0% vs 6.5%; *P* = 0.001), with involvement of all apical segments being more common (13.9% vs 3.7%). LV apical aneurysm at resolution was associated with a significantly increased risk of LVT recurrence during the follow-up period (Figures 1 and 3, Central Illustration, Supplemental Figure 2).

**Table 2:** Echocardiographic Characteristics at LVT Diagnosis, Stratified by LVT Recurrence

	Total (N = 252)	No LVT Recurrence (n = 216)	LVT Recurrence (n = 36)	HR (95% CI)	*P* Value
LVEF, mean (SD), %	30.5 (13.4)	30.5 (13.9)	30.5 (10.5)	1.00 (0.97-1.02)	0.87
Global hypokinesis, n (%)	67 (26.6)	60 (27.8)	7 (19.4)	0.62 (0.27-1.41)	0.26
LV apical wall motion abnormalities, n (%)	183 (72.6)	154 (71.3)	29 (80.6)	1.66 (0.73-3.80)	0.23
Hypokinesis, n (%)	10 (4.0)	9 (4.2)	1 (2.8)	0.73 (0.10-5.30)	0.75
Akinesis, n (%)	157 (62.3)	131 (60.6)	26 (72.2)	1.59 (0.77-3.30)	0.21
Dyskinesis, n (%)	4 (1.6)	4 (1.9)	0 (0)	-	**-**
Aneurysm, n (%)	12 (4.8)	10 (4.6)	2 (5.6)	1.42 (0.34-5.92)	0.63
1 segment, n (%)	0 (0)	0 (0)	0 (0)		
2 segments, n (%)	5 (2.0)	4 (1.9)	1 (2.8)		
3 segments, n (%)	1 (0.4)	1 (0.5)	0 (0)		
4 segments, n (%)	6 (2.4)	5 (2.3)	1 (2.8)		

LVEF = left ventricular ejection fraction; other abbreviation as in Table 1.

**Table 3:** Echocardiographic Characteristics at LVT Resolution, Stratified by LVT Recurrence

	Total (N = 252)	No LVT Recurrence (n = 216)	LVT Recurrence (n = 36)	HR (95% CI)	*P* Value
LVEF (%), mean (SD), %	38.7 (15.0)	40.0 (15.3)	34.0 (12.4)	0.98 (0.96-1.00)	0.04
Global hypokinesis, n (%)	49 (19.4)	43 (19.9)	6 (16.7)	0.72 (0.30-1.74)	0.47
LV apical wall motion abnormalities, n (%)	170 (67.5)	143 (66.2)	27 (75.0)	1.54 (0.72-3.27)	0.26
Hypokinesis, n (%)	29 (11.5)	29 (13.4)	0 (0)	-	**-**
Akinesis, n (%)	113 (44.8)	95 (44.0)	18 (50.0)	1.33 (0.69-2.56)	0.39
Dyskinesis, n (%)	5 (2.0)	5 (2.3)	0 (0)	-	**-**
Aneurysm, n (%)	23 (9.1)	14 (6.5)	9 (25.0)	3.82 (1.79-8.15)	0.001
1 segment, n (%)	2 (0.8)	1 (0.5)	1 (2.8)		
2 segments, n (%)	5 (2.0)	3 (1.4)	2 (5.6)		
3 segments, n (%)	3 (1.2)	2 (0.9)	1 (2.8)		
4 segments, n (%)	13 (5.2)	8 (3.7)	5 (13.9)		

Abbreviations as in Tables 1 and 2.

### Anticoagulation after LVT resolution

After LVT resolution, 196 patients (77.8%) were treated with OAC during the follow-up period, and this was not significantly different between those with LVT recurrence compared to those without recurrence (*P* = 0.19) (Supplemental Table 2). Similarly, the type of anticoagulation therapy used was not significantly different between groups; 31.7% of patients used vitamin K antagonists, 29.4% direct oral anticoagulants, and 16.7% switched between therapies.

However, among the 196 patients who were prescribed OAC following LVT resolution, only 126 (64.3%) met the criteria for “Uninterrupted OAC” for the duration of the follow-up period (ie, near continuous therapy aside from temporary interruption in therapy <14 consecutive days). The remaining 70 patients treated with OAC during the follow-up period either had prolonged interruption in therapy of at least 14 consecutive days or discontinued therapy during the follow-up period and were included in the “Interrupted/Discontinued OAC” group.

“Uninterrupted OAC” during the follow-up period was significantly more common among patients who did not experience LVT recurrence (54.7% vs 25.0%; *P* = 0.005) (Supplemental Table 3). Anticoagulation monotherapy (without antiplatelet therapy) was more common among patients without LVT recurrence (25.5% vs 2.8%; *P* = 0.002), whereas the concurrent use of anticoagulation and antiplatelet therapy (both single and dual antiplatelet) was not significantly different between groups (22.2% LVT recurrence vs 28.7% no LVT recurrence; *P* = 0.35). The use of antiplatelet monotherapy was also not significantly different between the groups (47.2% LVT recurrence vs 31.9% no recurrence; *P* = 0.17). However, “Interrupted/Discontinued OAC” without antiplatelet therapy following LVT resolution was significantly more common among patients with LVT recurrence (27.8% vs 13.9%; *P* = 0.02).

**Figure 2:**
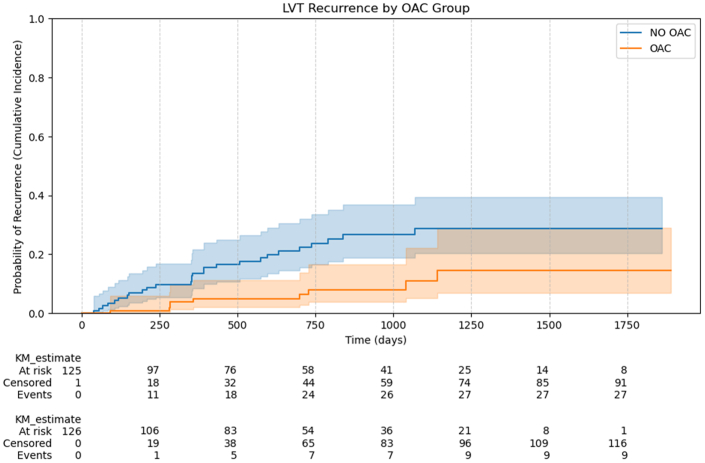
**Cumulative Incidence of LVT Recurrence, Stratified by Oral Anticoagulation Therapy** “Uninterrupted OAC” following LVT resolution was associated with a decreased risk of LVT recurrence during the follow-up period. OAC refers to “Uninterrupted OAC,” which was defined by near continuous oral anticoagulation therapy (aside from temporary interruption <14 consecutive days in duration) during the follow-up period. No OAC refers to “Interrupted/Discontinued OAC,” which was defined by prolonged interruption in oral anticoagulation therapy ≥14 consecutive days or permanent discontinuation in therapy during the follow-up period. OAC = oral anticoagulation; other abbreviations as in Figure 1.

**Figure 3:**
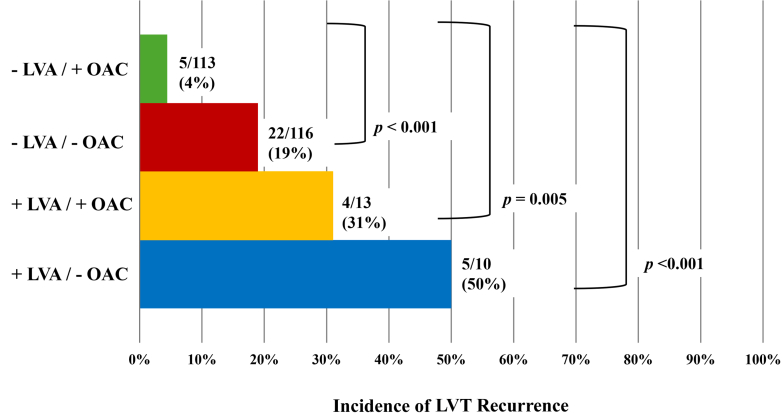
**LVT Recurrence Rate, Stratified by the Presence of Left Ventricular Apical Aneurysm and Oral Anticoagulant Therapy** An incremental increase in the risk of LVT recurrence was observed based on the presence of LV apical aneurysm and with “Interrupted/Discontinued OAC” after LVT resolution. LVA + was defined by LV apical aneurysm after initial LVT thrombus resolution, and LVA - was defined by no evidence of LV apical aneurysm on echocardiogram after initial LVT resolution. OAC + refers to “Uninterrupted OAC” following LVT resolution and was defined by near continuous anticoagulation during the follow-up period (aside from temporary interruption <14 consecutive days). OAC - refers to “Interrupted/Discontinued OAC,” which was defined by prolonged interruption in oral anticoagulation therapy ≥14 consecutive days or permanent discontinuation in therapy during the follow-up period. Abbreviations as in Figures 1 and 2.

“Uninterrupted OAC” following LVT resolution was associated with a decreased risk of LVT recurrence (Figures 2 and 3, Central Illustration, Supplemental Figure 2). A significant incremental increase in the risk of LVT recurrence was observed based on the presence of LV apical aneurysm and “Interrupted/Discontinued OAC” following LVT resolution. For the 113 patients without a LV apical aneurysm who were in the “Uninterrupted OAC” cohort, LVT recurrence occurred in 5 (4.4%). In contrast, for the 10 patients with a LV apical aneurysm who were in the “Interrupted/Discontinued OAC” cohort, 50.0% experienced LVT recurrence.

**Central Illustration:**
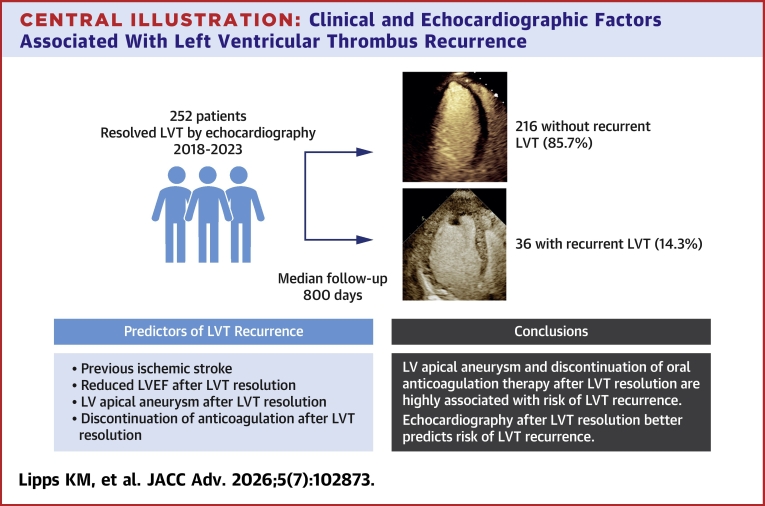
**Clinical and Echocardiographic Factors Associated With Left Ventricular Thrombus Recurrence** Among patients with resolved LVT, 14.3% experienced recurrent LVT, with higher rates observed in those with previous ischemic stroke and lower LVEF, LV apical aneurysm, and discontinuation of oral anticoagulation therapy after thrombus resolution. LV = left ventricular; LVEF = left ventricular ejection fraction; LVT = left ventricular thrombus.

### Clinical outcomes

During a median (Q1-Q3) follow-up of 800 (456-1205) days after initial LVT resolution, 36 of 252 (14.3%) patients had echocardiographic evidence of LVT recurrence. The composite outcome of stroke or systemic embolism occurred in 9 patients with a cumulative incidence of 4.7% (95% CI: 2.3-8.4); this was similar between patients with LVT recurrence (5.8%; 95% CI: 1.0-17.0) compared to those without (4.6%; 95% CI: 2.0-8.8). The individual components of stroke (5.8%; 95% CI: 1.0-17.0 LVT recurrence vs 3.4%; 95% CI: 1.2-7.4 no LVT recurrence) and systemic embolism (0% LVT recurrence vs 1.2% no LVT recurrence; 95% CI: 0.2-3.9) were also similar between groups. The cumulative incidence of major bleeding was 8.4% (95% CI: 4.2-16.3) in patients without LVT recurrence and 9.3% (95% CI: 3.0-26.3) in those with LVT recurrence. This difference did not reach statistical significance (HR = 1.89, 95% CI: 0.60-5.98; *P* = 0.28). The cumulative incidence of death following initial LVT resolution was 6.1% at 1 year, 21.7% at 3 years, and 34.1% at 5 years. Five-year mortality was 27.8% (95% CI: 20.8-36.6) in patients without LVT recurrence and 54.1% (95% CI: 31.1-80.4) in those with LVT recurrence. This difference also did not reach statistical significance (HR 1.17, 95% CI: 0.60-2.26; *P* = 0.65).

### Multivariable cox proportional HRs for LVT recurrence

In the Cox proportional hazards analysis, Model 1 showed that ICM was associated with an elevated risk of LVT recurrence, although this did not reach statistical significance (HR: 2.36; 95% CI: 0.95-5.80; *P* = 0.063). The presence of a LV apical aneurysm was associated with an increased recurrence risk (HR: 3.12; 95% CI: 1.43-6.84; *P* = 0.004), whereas higher LVEF (HR: 0.96 per 1% increase; 95% CI: 0.95-0.99; *P* = 0.007) and “Uninterrupted OAC” were associated with lower recurrence risk (HR: 0.27; 95% CI: 0.13-0.58; *P* < 0.001). In Model 2, “Uninterrupted OAC” remained independently associated with reduced risk of LVT recurrence (HR: 0.29; 95% CI: 0.13-0.63; *P* = 0.002), and a history of ischemic stroke was associated with increased risk (HR: 2.75; 95% CI: 1.22-6.17; *P* = 0.014). Hereditary thrombophilia was not significantly associated with recurrence risk (HR: 2.87; 95% CI: 0.68-12.1; *P* = 0.15). The results from the competing risk assessment were not appreciably different from the Cox proportional hazard model estimates (Table 4).

**Table 4:** Multivariable Cox Proportional Hazards and Competing Risk (Fine-Gray) Models for Predictors of LVT Recurrence

	HR, Cox Proportional Hazards Model (95% CI)	Subdistribution HR, Competing Risk (Fine-Gray) Model (95% CI)
Model 1		
ICM	2.36 (0.95-5.80)	2.13 (0.87-5.19)
LVEF	0.96 (0.95-0.99)	0.98 (0.96-0.99)
LV apical aneurysm	3.12 (1.43-6.84)	3.23 (1.48-7.06)
Uninterrupted OAC	0.27 (0.13-0.58)	0.30 (0.14-0.63)
Model 2		
Uninterrupted OAC	0.29 (0.13-0.63)	0.29 (0.13-0.64)
History of ischemic stroke	2.75 (1.22-6.17)	2.97 (1.29-6.81)
Hereditary thrombophilia^a^	2.87 (0.68-12.1)	3.27 (0.91-11.77)

ICM = ischemic cardiomyopathy; LV = left ventricular; OAC = oral anticoagulation; other abbreviations as in Tables 1 and 2.

a Includes Factor V Leiden, prothrombin gene mutation, Protein S deficiency, Protein C deficiency, and antithrombin deficiency.

## Discussion

This study evaluates the clinical and echocardiographic factors associated with LVT recurrence among patients with LVT resolution. We found that LVT recurrence occurred in 14.3% of patients and was more prevalent in ICM compared to other heart failure etiologies. The presence of lower LVEF and LV apical aneurysm by echocardiography at LVT resolution were better predictors of recurrence than these characteristics by echocardiography at initial LVT diagnosis. Following LVT resolution, “Uninterrupted OAC” was associated with a significantly lower risk of LVT recurrence. Stroke and systemic embolism, mortality, and bleeding were not significantly different between patients with LVT recurrence compared to those without, but this analysis had limited power to detect these differences due to sample size.

### Prevalence of LVT recurrence

Although LVT resolution is common in the contemporary era of anticoagulation therapy,^3^, ^4^, ^5^ recurrence rates are not insignificant.^12^, ^13^, ^14^, ^15^, ^16^, ^17^, ^18^, ^19^ Although literature regarding epidemiology and initial management of LVT is pervasive, the risk of LVT recurrence is not as well established. Furthermore, there is lack of consensus regarding duration of therapy in patients who experience LVT resolution with anticoagulation.^22^

To our knowledge, the current study is the largest to date to describe the association of clinical characteristics and imaging findings related to LV structure and function and LVT recurrence. Among our 252 patients with resolved LVT, recurrence rate was 14.3%, which is consistent with that reported in the literature (5.3-24.3%).^12^, ^13^, ^14^, ^15^, ^16^, ^17^, ^18^, ^19^ The lowest LVT recurrence rate (5.3%) was observed in a small retrospective cohort (n = 19) by Bawaskar et al;^18^ the lower recurrence rate compared to our population likely reflects the difference in population size and imaging modalities (CMR vs echocardiography). The highest recurrence rate was observed in a retrospective cohort (n = 115) by Zhou et al,^12^ which assessed LVT resolution and recurrence by echocardiography. In the largest previous cohort of patients with resolved LVT (n = 179), a recurrence rate of 11.2% was observed,^16^ similar to our findings

### Risk factors for LVT recurrence

Altogether, previous studies and our findings suggest that risk factors for LVT persist after thrombus resolution in many patients, thereby contributing to the risk of LVT recurrence. After acute myocardial infarction, myocardial remodeling is common,^23^^,^^24^ and previous studies evaluating LVT recurrence reported a prevalence of ICM of up to 75%.^16^^,^^18^ We observed a similar prevalence and found that ICM was more common in patients with LVT recurrence.

In addition, pathological remodeling can lead to LV aneurysm formation.^25^^,^^26^ In this study, LV apical aneurysm was observed in ∼5% of patients at LVT diagnosis and ∼10% at resolution. The increase in prevalence may be explained by the acuity of myocardial injury at LVT diagnosis, as pathological remodeling and LV aneurysm formation occur over weeks to months.^27^ Two previous studies have demonstrated that LV aneurysm at LVT diagnosis is a risk factor for recurrence.^12^^,^^14^ In this study we did not observe a significant difference in the prevalence of LV apical aneurysm between patients who ultimately developed LVT recurrence compared to those who did not when considering the echocardiogram at LVT diagnosis. Notably, the overall prevalence of LV apical aneurysm in our population was lower than observed in previous studies evaluating LVT recurrence.^12^^,^^14^^,^^18^ This could be secondary to our exclusion of patients with chronic LVT, which is associated with LV aneurysm,^18^ and because we only evaluated LV apical aneurysm, given this location is associated with the highest risk of LVT formation.^28^ From our data, we found that the presence of a LV apical aneurysm at LVT resolution was significantly more common among patients with LVT recurrence. This emphasizes the importance of interval imaging following acute myocardial injury to differentiate myocardial recovery from pathological remodeling.

LV systolic dysfunction is a well-established risk factor for LVT formation.^29^^,^^30^ In this study, all patients had evidence of LV systolic dysfunction, defined as LVEF <55% or regional wall motion abnormalities. The mean LVEF of patients at LVT diagnosis was similar between groups and was consistent with previous literature.^12^^,^^18^ However, at LVT resolution, the mean LVEF was significantly lower in patients who developed LVT recurrence. A higher prevalence of ST-segment elevation myocardial infarction in our population compared to that of previous studies^12^^,^^16^ may explain this finding, given the association with transmural infarction, pathological remodeling, and LV aneurysm formation.^25^^,^^26^

### Anticoagulation therapy following LVT resolution

Whether long-term anticoagulation reduces the risk of LVT recurrence among patients with resolved LVT is not well established. Two studies did not describe anticoagulation therapy following LVT resolution, thereby preventing evaluation of association of anticoagulation therapy with LVT recurrence.^17^^,^^18^ Four additional studies reported that all patients who experienced LVT recurrence had discontinued anticoagulation therapy.^13^, ^14^, ^15^^,^^19^ In three of these studies, anticoagulation therapy was not used in any patient following LVT resolution,^14^^,^^15^^,^^19^ and in one study, details regarding anticoagulation use in patients without LVT recurrence was lacking.^13^ Of the two studies that evaluated the association between anticoagulation therapy following LVT resolution and LVT recurrence, data are conflicting.^12^^,^^16^ Kim et al^16^ demonstrated that among patients with resolved LVT (*n* = 179), neither continuation nor duration of anticoagulation therapy were significant predictors of LVT recurrence. In contrast, Zhou et al^12^ (*n* = 115) observed that anticoagulation therapy following LVT resolution was associated with a lower rate of LVT recurrence; furthermore, among patients treated with anticoagulation, only those with LV aneurysm experienced recurrence.

Similarly, in our population, “Uninterrupted OAC” was associated with a significantly lower rate of LVT recurrence. However, a concerning finding was that even for patients without LV apical aneurysm who were in the “Uninterrupted OAC” group following LVT resolution, ∼5% experienced recurrence, which was not observed in the study by Zhou et al.^12^ The reason for this difference may be due to our exclusion of chronic LVT and the higher prevalence of ST-segment elevation myocardial infarction and ICM in our cohort.

Although “Uninterrupted OAC” was significantly more common among our patients who did not develop LVT recurrence, the use of antiplatelet therapy was similar between groups. This is not unexpected, given current guidelines recommend the use of anticoagulation therapy, rather than antiplatelet, for treatment and prevention of LVT.^22^

### Clinical implications

Despite the higher risk of LVT recurrence among patients in the “Interrupted/Discontinued OAC” group during the follow-up period, we did not observe significant differences in the incidences of stroke, systemic embolism, or mortality between those who experienced LVT recurrence and those who did not, although our study did not have the power to detect these differences. This contrasts with the study by Zhou et al,^12^ which found that patients with LVT recurrence experienced a significantly higher rate of major adverse cardiovascular events, including stroke and systemic embolism. The discrepancy in our results may be explained by the considerably higher mortality rate among our cohort (34.1% vs 3.5%), whereby our patients may have died before experiencing an embolic complication. Another potential explanation is more frequent imaging follow-up in our study that would allow for potential identification of recurrent LVT and reinitiation of anticoagulation before an embolic complication could occur.

Major bleeding was also not significantly different between patients who experienced LVT recurrence compared to those who did not, similar to what was described by Zhou et al.^12^ This is important, as the decision to continue anticoagulation therapy following LVT resolution must carefully consider whether bleeding risk outweighs that of LVT recurrence and associated embolic complications.

We observed an increase in LVT recurrence based on the presence of LV apical aneurysm and “Interrupted/Discontinued OAC” following LVT resolution, with the lowest rate occurring in those without LV apical aneurysm and who were treated with “Uninterrupted OAC” and the highest rate in those with LV apical aneurysm and who were in the “Interrupted/Discontinued OAC” group. Importantly, even among patients without LV apical aneurysm who were in the “Interrupted/Discontinued OAC” group following LVT resolution, nearly 20% experienced LVT recurrence. Consequently, continuation of OAC therapy following LVT resolution as a strategy to prevent LVT recurrence requires further study, particularly among those with significant LV systolic dysfunction, LV apical aneurysm, and low bleeding risk.

### Study Limitations

As with any retrospective study, causal nature cannot be established, and confounding variables may have influenced outcomes despite careful analysis. The NLP algorithm was designed to maximize specificity rather than sensitivity; consequently, less certain (mass vs thrombus) cases of LVT may have been missed, thus biasing our cohort toward more clear or severe cases of LVT. Due to the focused evaluation of this study, we included only patients with resolved LVT, excluding 19.2% of patients with persistent LVT, and therefore our findings are only pertinent to those who survive to time of LVT resolution.

We relied on clinical echocardiographic reports (rather than independent, centralized review of images) to determine LVT resolution and characteristics of LV structure and systolic function—at LVT diagnosis and at resolution—which is a methodological limitation given interindividual variability in echocardiogram interpretation. However, this is reflective of real-world clinical practice. In addition, lack of universal use of ultrasound enhancing agent may have contributed to misclassification bias, whereby LVT which decreased in size during follow-up may have been missed and mislabeled as recurrent LVT if it was identified later. However, most echocardiograms used ultrasound enhancing agent. Similarly, we did not evaluate the association of other imaging modalities to identify LVT or characterize LV structure or function. We acknowledge that CMR is superior for tissue characterization and distinguishing thrombus from other intracardiac mass types as well as delineating myocardial borders and identifying myocardial scar and LV aneurysm formation. Finally, we did not have data regarding the frequency of echocardiographic examinations in our cohort, which may have created detection bias where patients with more severe cardiomyopathy received more frequent imaging and therefore earlier or more reliable identification of LVT recurrence.

Our results were also subject to confounding by indication, whereby patients in the “Interrupted/Discontinued OAC” group may have been at higher risk of LVT recurrence, but uninterrupted anticoagulation therapy was contraindicated due to underlying critical illness, bleeding risk, or frailty. These covariates were not adjusted for in the Cox model. Adding to this, we grouped patients treated with either vitamin K antagonists or direct oral anticoagulants together for the purposes of analysis, which may have influenced the association between anticoagulation use and outcomes, particularly since time in therapeutic range for vitamin K antagonists was not available. Multiple comparisons were made between clinical and echocardiographic characteristics and the primary outcome of LVT recurrence, which increases the risk of a Type 1 error. Although we performed a multivariable Cox proportional hazard regression analysis to identify independent predictors of LVT recurrence, univariable predictors were distributed across two Cox models to limit overfitting in the setting of a low incidence of LVT recurrence. In addition, we did not consider other predictors, which may have been important, including the use of ultrasound enhancing agent, etiology of acute cardiac disease and cardiomyopathy, and for patients with ICM, duration between myocardial infarction and LVT diagnosis, infarct territory, and infarct size or scar burden. Finally, although we identified significant differences in LVT recurrence based on the use of anticoagulation therapy, our study may have been underpowered to detect differences in stroke, systemic embolism, and mortality.

### Future directions

Despite limitations, our study highlights the need for further research regarding the role of long-term OAC therapy after LVT resolution, especially for patients with ICM, reduced LVEF, or LV apical aneurysm, who are at low bleeding risk.

## Conclusions

Among patients with LVT resolution, 14% experienced LVT recurrence, and the incidence was higher in those with previous ischemic stroke and those with reduced LVEF or LV apical aneurysm identified by echocardiography at the time of LVT resolution. LVT recurrence was also higher in patients in the “Discontinued/Interrupted OAC” group following LVT resolution. Given risk of LVT recurrence in all groups, indefinite OAC therapy may be considered for all patients with previous LVT, particularly among those with low bleeding risk, although whether this translates into clinically meaningful outcomes remains uncertain.

Perspectives **COMPETENCY IN MEDICAL KNOWLEDGE AND PATIENT CARE:** Among patients who experience LVT resolution while on OAC, LVT recurrence occurred in 14% with significantly higher rates observed in patients with lower LVEF at the time of LVT resolution, LV apical aneurysm, and those who discontinue OAC after resolution of initial LVT.

**TRANSLATIONAL OUTLOOK:** There is a need for continued research surrounding late clinical events and the optimal anticoagulation strategies in patients with resolved LVT.

## Funding support and author disclosures

This publication was made possible by the Mayo Clinic Clinical and Translational Science Award through grant number UL1TR002377 from the 10.13039/100006108National Center for Advancing Translational Sciences, a component of the National Institutes of Health. The authors have reported that they have no relationships relevant to the contents of this paper to disclose.
