# Supplementary material for: Clinical and Echocardiographic Factors Associated With Left Ventricular Thrombus Recurrence
Source: JACC Adv. 2026 Jun 17;5(7):102873. doi: 10.1016/j.jacadv.2026.102873 (PMC13311162; doi:10.1016/j.jacadv.2026.102873)
Supplement: Supplemental_Material [file mmc1.docx]

# Clinical and Echocardiographic Factors Associated with Left Ventricular Thrombus Recurrence

Kirsten M. Lipps, M.D.^a^; Hossam Elbenawi, M.D.^a^; Samuel Heller Jr., M.D.^b^; Robert D. McBane, M.D.^a^; Waldemar E. Wysokinski, M.D., Ph.D.^a^; Ana I. Casanegra, M.D., M.S.^a^; Stanislav Henkin, M.D.^a^; Thom W. Rooke, M.D.^a^; Paul W. Wennberg, M.D.^a^; David A. Liedl, M.S.^a^; Raymond C. Shields, M.D.^a^; Damon E. Houghton, M.D., M.S.^a,c^

a Division of Vascular Medicine, Department of Cardiovascular Medicine, Mayo Clinic, 200 First St. SW, Rochester, Minnesota 55905, United States

b Second Faculty of Medicine, Charles University, V Úvalu 84, 150 06 Prague 5, Czech Republic

c Division of Hematology, Department of Internal Medicine, Mayo Clinic, 200 First St. SW, Rochester, Minnesota 55905, United States

# Table of Contents

Supplemental Figure 1. Criteria for Patient Selection Page 3

Supplemental Figure 2. Kaplan Meier Curve of LVT Recurrence, Stratified

by Apical Left Ventricular Aneurysm at Resolution and Continuation of

Oral Anticoagulation Therapy Page 4

Supplemental Table 1. Etiology of Cardiomyopathy, Stratified by

LVT Recurrence Page 6

Supplemental Table 2**.** Use of Oral Anticoagulation Therapy Following

LVT Resolution, Stratified by LVT Recurrence Page 7

Supplemental Table 3. Uninterrupted vs Interrupted/Discontinued Therapy

and Antiplatelet Therapy after LVT Resolution, Stratified by LVT Recurrence Page 9

# Supplemental Figure 1. Criteria for Patient Selection

**
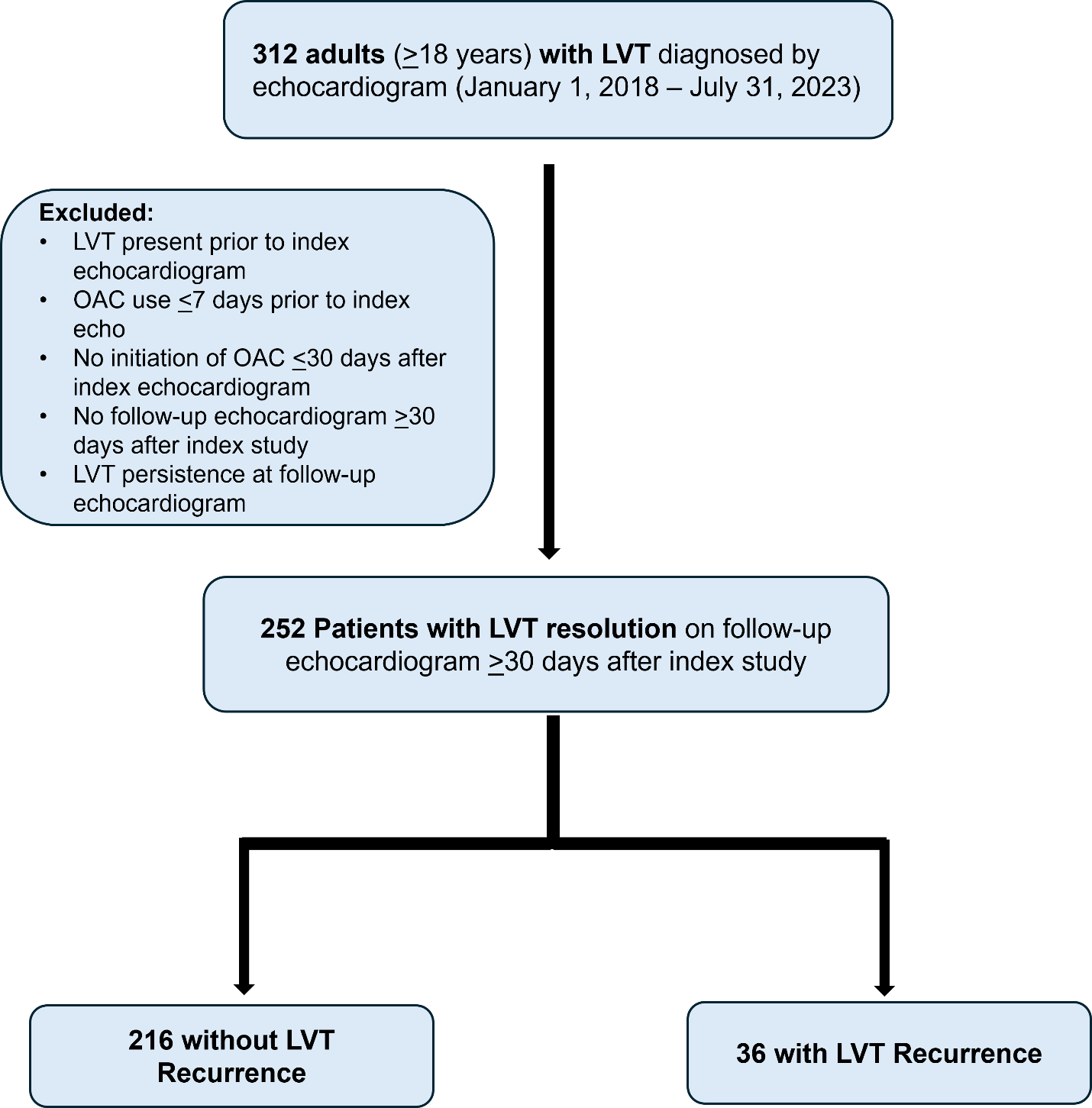
**

Legend: Criteria for patient selection for inclusion in analysis.

Abbreviations: LVT, left ventricular thrombus; OAC, oral anticoagulation therapy

# Supplemental Figure 2. Kaplan Meier Curve of LVT Recurrence, Stratified by Apical Left Ventricular Aneurysm at Resolution and Continuation of Oral
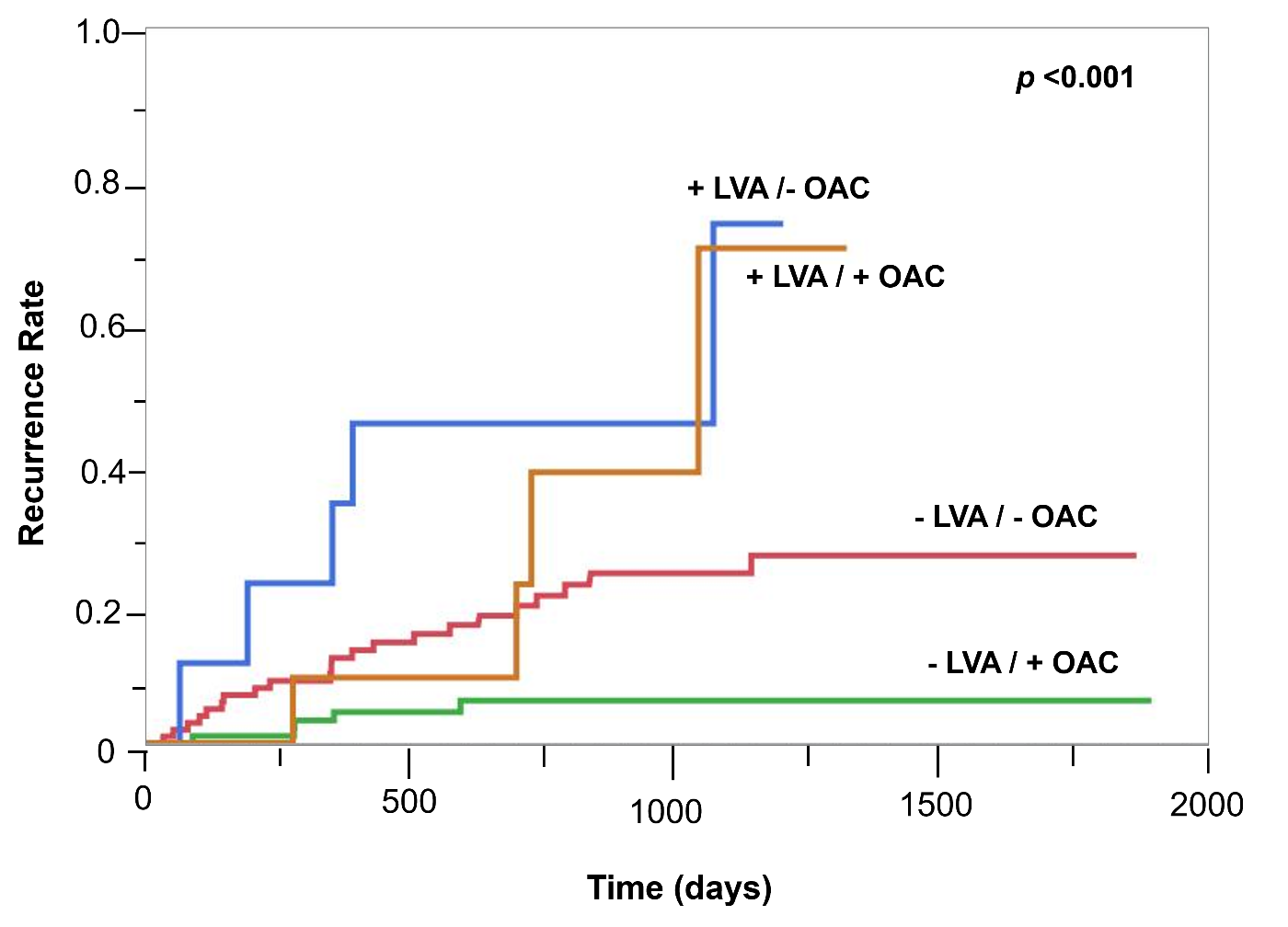
 Anticoagulation Therapy

Legend: An incremental increase in LVT recurrence rate was observed based on the presence of left ventricular aneurysm (LVA +) and discontinuation or interruption of oral anticoagulation therapy (OAC -) after LVT resolution. “Uninterrupted Oral Anticoagulation” was defined by near continuous use of oral anticoagulation therapy during the follow-up period (aside from temporary interruption <14 consecutive days in duration) and “Interrupted/Discontinued Oral Anticoagulation” was defined by prolonged interruption in therapy >14 consecutive days or permanent discontinuation in therapy during the follow-up period. Anticoagulation therapy was evaluated from the time of index LVT resolution until death or September 2024 (whichever occurred first).

Abbreviations: LVA, left ventricular aneurysm (+ LVA, LVA present; – LVA, no LVA); LVT, left ventricular thrombus; OAC, oral anticoagulation therapy (+ OAC, “Uninterrupted Oral Anticoagulation”; – OAC, “Interrupted/Discontinued Oral Anticoagulation”)

# Supplemental Table 1. Etiology of Cardiomyopathy, Stratified by LVT Recurrence

| **Type of Cardiomyopathy** | **Total**  **(n = 252)** | **No LVT**  **Recurrence**  **(*n* = 216)** | **LVT**  **Recurrence**  **(*n* = 36)** | ***P-*value** |
| --- | --- | --- | --- | --- |
| Acute myocardial disease ^a^, *n* (%) | 13 (5.2) | 13 (6.0) | 0 (0) | 0.040 |
| Ischemic, *n* (%) | 162 (64.3) | 132 (61.1) | 30 (83.3) | 0.003 |
| Dilated non-ischemic, *n* (%) | 54 (21.4) | 48 (22.2) | 6 (16.7) | 0.34 |
| Stress-induced, *n* (%) | 8 (3.2) | 8 (3.7) | 0 (0) | 0.10 |
| Hypertrophic, *n* (%) | 2 (0.8) | 2 (0.9) | 0 (0) | 0.42 |
| Peripartum, *n* (%) | 1 (0.4) | 1 (0.5) | 0 (0) | 0.57 |
| Sarcoid, *n* (%) | 2 (0.8) | 2 (0.9) | 0 (0) | 0.42 |
| Restrictive, *n* (%) | 1 (0.4) | 1 (0.5) | 0 (0) | 0.56 |
| Amyloid, *n* (%) | 1 (0.4) | 1 (0.5) | 0 (0) | 0.57 |
| Tachycardia-mediated, *n* (%) | 2 (0.8) | 2 (0.9) | 0 (0) | 0.42 |
| Unknown, *n* (%) | 6 (2.4) | 6 (2.8) | 0 (0) | 0.16 |

Abbreviations: LVT, left ventricular thrombus

^a^ Includes acute myocardial infarction and acute myocarditis in patients with absence of chronic cardiomyopathic condition.

# **Supplemental Table 2. Use of Oral Anticoagulation Therapy Following LVT Resolution, Stratified by LVT Recurrence**

| **Therapy** ^a^ | **Total**  **(n = 252)** | **No LVT Recurrence**  **(n = 216)** | **LVT Recurrence**  **(n = 36)** | ***P-*value** |
| --- | --- | --- | --- | --- |
| Anticoagulation, *n* (%) | 196 (77.8) | 171 (79.2) | 25 (69.4) | 0.19 |
| DOAC, *n* (%) | 74 (29.4) | 64 (29.6) | 10 (27.8) | 0.82 |
| VKA, *n* (%) | 80 (31.7) | 68 (31.5) | 12 (33.3) | 0.82 |
| Switch ^b^, *n* (%) | 42 (16.7) | 39 (18.1) | 3 (8.3) | 0.11 |
| No anticoagulation ^c^, *n* (%) | 56 (22.2) | 45 (20.8) | 11 (30.5) | 0.20 |

Abbreviation: DOAC, direct oral anticoagulant; LVT, left ventricular thrombus; VKA, vitamin K antagonist.

^a^ Defined as any use of oral anticoagulation therapy and included patients characterized as “Uninterrupted Oral Anticoagulation,” defined by near continuous use of oral anticoagulation therapy during the follow-up period (aside from temporary interruption <14 consecutive days in duration) and those classified as “Interrupted/Discontinued Oral Anticoagulation,” defined by prolonged interruption in therapy >14 consecutive days or indefinite discontinuation of therapy during the follow-up period. Anticoagulation therapy was evaluated from the time of index LVT resolution until death or September 2024 (whichever occurred first).

^b^ Included patients who switched between DOAC and VKA during the follow-up period after LVT resolution.

^c^ These patients did not use anticoagulation therapy at any time following resolution of the initial LVT until the end of the follow-up period.

**Supplemental Table 3. Uninterrupted vs Interrupted/Discontinued Anticoagulation Therapy and Antiplatelet Therapy after LVT Resolution, Stratified by LVT Recurrence**

| **Therapy** | **Total**  **(*n* = 252)** | **No LVT**  **Recurrence**  **(*n* = 216)** | **LVT**  **Recurrence**  **(*n* = 36)** | **Hazard Ratio (95% CI)** | ***P-*value** |
| --- | --- | --- | --- | --- | --- |
| Uninterrupted oral anticoagulation ^a^, *n* (%) | 126 (50.0) | 117 (54.7) | 9 (25.0)7 | 0.34 (0.16–0.72) | 0.005 |
| Anticoagulation only, *n* (%) | 56 (22.2) | 55 (25.5) | 1 (2.8) | 0.56 (0.28–1.15) | 0.11 |
| Anticoagulation + antiplatelet, *n* (%) | 70 (27.8) | 62 (28.7) | 8 (22.2) | 0.69 (0.31–1.51) | 0.35 |
| Anticoagulation + SAPT, *n* (%) | 62 (24.6) | 56 (25.9) | 6 (16.7) | 0.57 (0.24–1.36) | 0.20 |
| Anticoagulation + DAPT, *n* (%) | 8 (3.2) | 6 (2.8) | 2 (5.6) | 1.75 (0.42–7.29) | 0.44 |
| Antiplatelet only, *n* (%) | 86 (34.1) | 69 (31.9) | 17 (47.2) | 1.58 (0.82–3.05) | 0.17 |
| Interrupted or discontinued oral anticoagulation  and no antiplatelet ^b^, *n* (%) | 40 (15.9) | 30 (13.9) | 10 (27.8) | 2.34 (1.13–4.87) | 0.02 |

Abbreviations: DAPT, dual antiplatelet therapy; DOAC, direct oral anticoagulant; LVT, left ventricular thrombus; SAPT, single antiplatelet therapy; VKA, vitamin K antagonist

^a^ “Uninterrupted Oral Anticoagulation” was defined by near continuous use of oral anticoagulation therapy during the follow-up period (aside from temporary interruption <14 consecutive days in duration), from the time of index LVT resolution death or September 2024 (whichever occurred first).

^b^ “Interrupted/Discontinued Oral Anticoagulation” was defined by prolonged interruption in therapy >14 consecutive days or indefinite discontinuation of therapy during the follow-up period, from the time of index LVT resolution until death or September 2024 (whichever occurred first).
